# Dewey’s Modification for Angle’s Class I Malocclusion: Revisited

**DOI:** 10.7759/cureus.53490

**Published:** 2024-02-03

**Authors:** Nishu Agarwal, Pallavi Daigavane, O P Kharbanda, Priyanka Niranjane, Sumukh Nerurkar, Mrudula Shinde

**Affiliations:** 1 Orthodontics and Dentofacial Orthopaedics, Sharad Pawar Dental College and Hospital, Datta Meghe Institute of Higher Education and Research, Wardha, IND; 2 Orthodontics and Dentofacial Orthopaedics, Ramaiah Faculty of Dental Sciences, Bangalore, IND

**Keywords:** malocclusion management, angle’s classification, classification, dewey’s modification, class i malocclusion

## Abstract

Introduction

Classification is a crucial communication tool between dental school professors and students, between practitioners, and between practitioners and insurance companies or government bureaucracies. The management of patients is significantly impacted by classification. Once a patient has been categorized, the practitioner will frequently use treatment strategies corresponding to that classification. The classification used by orthodontists most frequently is Dewey's version of Angle's categorization. To date, the shortcomings of Dewey’s modification were not mentioned in the literature. Various other malocclusions are still not included in this classification system that was modified by Dewey.

Aim

The aim of this study was to re-evaluate and re-establish Dewey’s modification for class I malocclusion for the various other types of malocclusions that are not included in the classification system.

Material and method

An observational study was carried out on a total of 600 patients in the Department of Orthodontics. The study duration was eight months. The photographic method was used for the evaluation of the malocclusion. Photographs were taken and clinical evaluation was done of the selected cases. The type of malocclusion was observed and recorded.

Result

The results showed that other types of malocclusions other than that of Dewey's modification are observed in the population. A total of 4% of the population was affected with single tooth crossbite and 5% were affected in more than one tooth. A total of 1% of the population was affected with single-tooth scissor bites, and in 2%, more than one tooth was involved. In 9% of the population, single-tooth rotations were present, whereas in 6%, more than one tooth was involved. A total of 35% of the population showed other types of malocclusions.

Conclusion

To conclude, various other malocclusions are present in the population suggesting a lacuna in Dewey's modification. Hence, there was a need to revisit.

## Introduction

Malocclusion compromises the health of oral tissues and also can lead to psychological and social problems [1]. Well-aligned teeth not only contribute to the health of the oral cavity and stomatognathic system but also influence the personality of the individual [2]. Because most malocclusions are morphogenetic, we may be certain that this dentofacial issue will require the best dental care for a very long time [3]. In many regions of the world, numerous organized population surveys have been conducted to assess the pattern and distribution of malocclusion [4]. For every target group in a community, a systematic and well-organized dental care program needs some basic information, like the pattern of the condition. It would be advantageous to gather additional data about patients given the growing interest in the early detection and treatment of malocclusion and the associated emphasis on preventive measures [5].

Between dental school professors and students, between practitioners, and between practitioners and insurance companies or government bureaucracies, classification is a crucial communication tool. The management of patients is significantly impacted by classification. Once a patient has been categorized, the practitioner will frequently use treatment strategies corresponding to that classification.

Even though there is no one best approach to categorize malocclusions, Angle's classification (first in 1898) [6,7] is the most widely used and widely recognized. Angle's categorization has frequently been utilized in determining the prevalence of malocclusions in communities in addition to its clinical applications. Given that both clinicians and epidemiologists frequently utilize Angle's classification, it is surprising that there haven't been many studies into its dependability. His classification of malocclusion was published in Dental Cosmos [8]. Angle's categorization has drawn criticism from several sites. First, some have opposed Angle by creating their own classification schemes; Dewey [2,5,7-10] is among the most known of these. Dewey modified Class I malocclusion with five types and Class III malocclusion with three types [10].

The most frequently used classification by orthodontists is Dewey's version of Angle's categorization. To date, the shortcomings of Dewey’s modification were not mentioned in the literature. Various other malocclusions are still not included in this classification system that was modified by Dewey. Those malocclusions include single tooth malposition like transposition of teeth, rotations, intrusions, deep bite, open bite, generalized spacing without proclination (i.e., upright on the alveolar bone), rectroclination of upper anterior with class I molar relationship in skeletal class I cases, and arch length tooth size discrepancy. The attempt was thus to fulfill the requirements and revisit Dewey’s modification to Angle’s classification for class I. To identify any potential irregularities in the developing dentofacial complex and suggest a modification to Dewey's classification, the goal of this study was to estimate the distribution and pattern of malocclusion in patients presenting to the Department of Orthodontics and Dentofacial Orthopaedics.

## Materials and methods

In the conducted observational study within the Department of Orthodontics, Sharad Pawar Dental College, Datta Meghe Institute of Medical Sciences, Wardha, India, meticulous attention was given to ethical considerations as evidenced by the approval from the Institutional Ethics Committee, marked by the Ethical Clearance Number DMIMS(DU)/IEC/2022/873. The study aimed to investigate class I malocclusion cases and cases with the presence of all permanent teeth, thereby setting clear inclusion criteria. To maintain the specificity of class I malocclusion, certain groups were intentionally excluded, such as patients with congenital deformities, class II and class III malocclusion cases, cleft lip and palate cases, and individuals lacking first permanent molars.

A convenient sampling method was employed to address the absence of similar articles in the existing literature, ensuring practicality in participant selection. This approach allowed the researchers to work with available and accessible subjects within the study's context. Prior to participation, explicit consent was obtained from all patients, emphasizing the ethical principle of informed consent. A convenient sampling method was used as this kind of study has not been done before in the literature and a statistical method could not be applied for sample size calculation.

The study's sample size comprised a robust cohort of 600 patients, all presenting with class I malocclusion cases, and these participants were drawn from the Outpatient Department (OPD). Employing a comprehensive approach to data collection, the researchers utilized the photographic method to capture detailed records of all patients under evaluation. After photographic documentation, clinical evaluations were conducted on the selected cases, enhancing the depth of the study's findings.

Over the course of eight months, the observational study unfolded, allowing for a thorough examination of the selected cases. The duration of the study is noteworthy as it reflects the timeframe within which data collection and evaluations transpired. The primary focus during the study was the meticulous observation and recording of the type of malocclusion exhibited by each participant, contributing to a comprehensive understanding of class I malocclusion cases within the studied population. This methodological approach, characterized by ethical diligence, a suitable sampling strategy, and a multifaceted data collection process, positions the study to yield valuable insights into the orthodontic landscape.

## Results

Malocclusions other than that of Dewey's modification are observed in the population. Spacing in the upper arch was seen in 11% of the population whereas in the lower arch, only 8% of the population showed spacing. A total of 10% of the population showed retroclined upper anterior with class I malocclusion (Table 1).

**Table 1:** Various malocclusions seen in upper and lower jaws

S. No.	Malocclusion		Absent	Present
1.	Spacing	Upper	534(89%)	66(11%)
		Lower	552(92%)	48(8%)
		Both	564(94%)	36(6%)
2.	Transposed	Upper	594(99%)	6(1%)
		Lower	600(100%)	0(0%)
3.	Retroclined incisors	Upper	540(90%)	60(10%)

A total of 4% of the population was affected with single-tooth crossbite and 5% was affected in more than one tooth. A total of 1% of the population was affected with single-tooth scissor bite whereas in 2%, it involved more than one tooth. In 9% of the population, single tooth rotations were present whereas in 6%, more than one tooth was involved. Transposition was present in 1% of the population in the upper arch whereas it was absent in the lower arch. A total of 35% of the population showed other types of malocclusions (Table 2).

**Table 2:** Various malocclusions seen in one or more teeth

S.no.	Malocclusion		Absent	one tooth affected	>one tooth affected
1.	Crossbite		546(91%)	24(4%)	40(5%)
2.	Scissor bite		582(97%)	6(1%)	12(2%)
3.	Missing	Anterior	588(98%)	0(0%)	12(2%)
		Premolar	594(99%)	6(1%)	0(0%)
		Molar	594(99%)	6(1%)	0(0%)
4.	Rotated		510(85%)	54(9%)	36(6%)

In Angle's class I molar, the mandibular first permanent molar's mesiobuccal groove encloses the maxillary first permanent molar's mesiobuccal cusp. Based on these values, a new classification system is devised, which includes three more types in addition to the original five types of Dewey's modification, which is mentioned in Table 3.

**Table 3:** Dewey's modification for Angle’s Class I malocclusion revisited

Type	Description
Type 6	Generalized spacing in the upper and lower arch with upright anterior on the alveolar bone due to arch size tooth length discrepancy
Type 7	Retroclined upper anterior with mild grade malocclusion in lower anterior
Type 8	Single tooth malocclusion

## Discussion

Various types of malocclusions have been given to date. Some of them are Ackerman and Profitt's classification [11], such as Katz's classification [12], and Lischer's classification [13]. The most common of them is Angle's classification modified by Dewey. Angle's categorization has drawn criticism from several sites. First, some have opposed Angle by creating their classification schemes, with Dewey standing out among them. A study on Angle's classification of malocclusion for the assessment of reliability was conducted by Gravely et al. They discovered that Angle's technique cannot be used very reliably [1]. A study was done by Yadav et al. to categorize ambiguous malocclusions that are in the middle of the spectrum and cannot be classified as full cusp classes I, II, or III [3].

According to Dewey, the criticism for the malocclusion arose because clinicians have based the classification upon one or two teeth instead of the entire arch. To date, Dewey’s modification of Angle is the most widely accepted classification among orthodontists. However various other malocclusions are not present in this classification system by Dewey. In a study conducted by Balina et al. [14], some patients could not be included in the modification given by Dewey because they failed to adhere to the characteristic features given by Dewey in his original classification. Hence, there was a need to revisit.

Out of the 600 cases included in this study, spacing in the upper arch was seen in 11% of the population whereas in the lower arch, only 8% of the population showed spacing. A total of 10% of the population showed retroclined upper anterior with class I malocclusion. These various malocclusions discussed above could not find a place in Dewey’s classification system; therefore, a new classification system that could incorporate these malocclusions was devised.

Despite the valuable insights gained from this study into the diverse spectrum of malocclusions and the introduction of a novel classification system, certain limitations must be acknowledged. The foremost constraint is the relatively small sample size utilized, which raises concerns regarding the generalizability of the findings. The study's focus on a localized area further restricts the extrapolation of results to broader populations, potentially neglecting regional variations in dental and craniofacial characteristics influenced by genetic, environmental, and cultural factors. Additionally, the lack of demographic diversity within the sample may limit the applicability of the proposed classification system across various age groups, ethnicities, and socio-economic backgrounds.

## Conclusions

In this comprehensive study, an extensive examination of malocclusions within the population has revealed a diverse range of dental irregularities. Among these, notable malocclusions include spacing with anterior teeth, retroclined anteriors, single-tooth crossbite, transposition of teeth, rotations, and an array of other types, emphasizing the multifaceted nature of orthodontic issues. Recognizing the need for a more nuanced and inclusive classification system, this research has undertaken the ambitious task of not only addressing individual tooth malpositions but also considering the arch as a whole. The proposed classification system aims to streamline communication within the field of orthodontics, offering practitioners a standardized framework to convey and categorize malocclusions effectively. This system is designed to enhance precision in diagnosis, treatment planning, and communication among orthodontic professionals, ultimately contributing to improved patient care.

In summary, this study advocates for a paradigm shift in the classification of malocclusions, emphasizing the significance of both individual teeth and the overall dental arch. By doing so, it aspires to foster a more comprehensive understanding of malocclusions, paving the way for enhanced orthodontic diagnosis and treatment strategies.
